# Biochemical Characterization of a Thiol-Activated, Oxidation Stable Keratinase from *Bacillus pumilus* KS12

**DOI:** 10.4061/2010/132148

**Published:** 2010-07-15

**Authors:** Rinky Rajput, Richa Sharma, Rani Gupta

**Affiliations:** Department of Microbiology, University of Delhi, South Campus, New Delhi 110021, India

## Abstract

An extracellular keratinase from *Bacillus pumilus* KS12 was purified by DEAE ion exchange chromatography. It was a 45 kDa monomer as determined by SDS PAGE analysis. It was found to be an alkaline, serine protease with pH and temperature optima of 10 and 60°C, respectively. It was thiol activated with two- and eight-fold enhancement in presence of 10 mM DTT and *β*-mercaptoethanol, respectively. In addition, its activity was stimulated in the presence of various surfactants, detergents, and oxidizing agents where a nearly 2- to 3-fold enhancement was observed in presence of H_2_O_2_ and NaHClO_3_. It hydrolyzed broad range of complex substrates including feather keratin, haemoglobin, fibrin, casein,and *α*-keratin. Analysis of amidolytic activity revealed that it efficiently cleaved phenylalanine → leucine → alanine- *p*-nitroanilides. It also cleaved insulin B chain between Val^2^- Asn^3^, Leu^6^-Cys^7^ and His^10^-Leu^11^ residues.

## 1. Introduction

Keratinases [3.4.21/24/99.11] are produced by a large number of bacteria, actinomycetes, and fungi, with unique ability to act on “hard to degrade” keratins which are found in bulk in the environment in the form of feather, nail, hair, hoof and so forth [1, 2]. By virtue of this ability, they are being exploited in various biotechnological applications in the sectors of ecologically friendly leather processing, nutritional improvement of waste feather for livestock feed, and production of protein hydrolysates from keratinous waste materials [2, 3]. In addition to this, they are also considered as desirable detergent additives which could replace the traditional proteases for removal of keratinous soils that are often encountered in laundry, such as collar washings to remove scurf and for eliminating horny epithelial cells adhered to textile fibers [1, 2]. To get significant importance as detergent additives, they should be robust in terms of their compatibility towards oxidizing agents, detergents, and surfactants and stability under high temperature and alkaline pH [2, 4]. However, oxidation stability is not a commonly encountered characteristic among well known detergent compatible proteases and is generally induced in them by site-directed mutagenesis and protein engineering [5, 6]. Thus, keratinases with robust characteristics like oxidation stability, alkaline stability, detergent compatibility, and temperature tolerance can fetch a good market among detergent proteases. Here we report the detailed characterization of a detergent compatible and oxidation stable keratinase from *Bacillus pumilus* KS 12.

## 2. Materials and Method

### 2.1. Bacterial Strain

A feather degrading strain of* Bacillus pumilus *KS12 isolated from garden soil of South Campus, New Delhi was used for the present study. Stock cultures of the strain were maintained in 50% (v/v) glycerol and stored at −80°C. The complete 16S rDNA sequence of the present strain has been submitted to the Gen Bank database (Accession no. EU874879).

### 2.2. Source of Keratin Substrate

Chicken feathers for the keratinase production were obtained from local poultry plants. Feathers were washed with distilled water and finally autoclaved at 15psi. The autoclaved feathers were dried in an oven.

### 2.3. Keratinase Production

Feather soy flour medium containing following constituents (g/L): chicken feather 5.0, glucose 5.0, soy flour 5.0, KH_2_PO_4 _1.0, K_2_HPO_4 _3.0, and MgCl_2 _5 mM, (pH 7.0) was used for keratinase production. Production was carried out in 250 mL Erlenmeyer flask containing 50 mL\ medium for 24 h at 37°C, 200 rpm in an incubator shaker (New Brunswick Scientific, Edison NJ, USA).

### 2.4. Keratinase Activity and Protein Estimation

The keratinase activity was determined as described by Dozie et al. [7]. The assay mixture containing 1 mL of appropriately diluted enzyme, 4 mL glycine-NaOH buffer (50 mM, pH9.0), and 20 mg of chicken feather was incubated at 70°C for 1 h. The reaction was terminated by adding 4 mL of 5% (w/v) trichloroacetic acid. Insoluble residues were removed by filtration through glass wool, and the filtrate was centrifuged at 5000 × g for 10 min. 

Appropriate controls were also set up. Proteolytic products in the supernatant were determined by absorbance at 280 nm against controls. An increase in absorbance of 0.01 was considered as 1 U enzyme activity. The protein content was estimated by the method of Lowry [8] using BSA as standard in the concentration range 100–1000 *μ*g/mL.

### 2.5. Purification of Keratinase

One gram of the lyophilized enzyme was dissolved in 5 mL of 10 mM Tris-HCl buffer (pH 8) and loaded on DEAE-Sepharose column (Pharmacia Biotechnology Upsala, Sweden) equilibrated with 10 mM Tris-HCl buffer (pH8). The column was washed with the same buffer, and 15 mL fraction was collected at a flow rate of 1 mL/min. Bound protein was eluted in a linear salt gradient (0.1 M–3 M NaCl). Protein elution was monitored at 280 nm, and keratinolytic activity and protein were determined as described earlier.

### 2.6. SDS-PAGE and HPLC Analysis

Sodium dodecyl sulphate-polyacrylamide gel electrophoresis was carried out using the method of Laemmli [9] to check the purity of purified fraction,* and its *purity was confirmed by HPLC using C18column (Shimadzu, Japan) and acetonitrile: water (90 : 10) as mobile phase with flow rate of 0.5 ml/min. The protein was detected at A_280_ using UV detector.

### 2.7. Zymogram

The activity staining of purified keratinase was performed by reactivating 10% SDS-PAGE. After electrophoresis, the gel was soaked for 20 min in 2.5% (v/v) Triton X-100 with constant shaking to remove SDS. The gel was washed thrice with glycine-NaOH buffer (100 mM, pH 10.0) to remove Triton X-100. The gel was overlaid onto a 1% casein agar plate prepared in glycine-NaOH buffer (100 mM, pH 10.0). Enzyme activity was visualized by incubating the gel for 4 h at 50°C. The reaction was stopped by flooding with 5% (w/v) TCA. The band of purified keratinase appeared as clear zone against a white background.

### 2.8. Biochemical Properties of Keratinase

#### 2.8.1. Effect of pH and Temperature on the Activity and Stability of Keratinase

The effect of pH was studied by carrying out the keratinase assay in the pH range of 3.0–12.0 using 50 mM buffers: pH 3.0–6.0 (citrate phosphate buffer), pH 7.0-8.0 (sodium phosphate buffer), pH 9.0-10.0 (glycine-NaOH buffer), pH 11.0 (phosphate hydroxide buffer), and pH 12.0 (hydroxide-chloride buffer). Similarly, the effect of temperature on keratinase activity was determined by incubating the enzyme at temperatures ranging from 4–80°C at pH 10. Activity was expressed as percentage relative activity with respect to maximum activity, which was considered as 100%. 

The pH stability was determined by preincubating the purified keratinase in buffers of varying pH (3.0–12.0) for 2 h at room temperature, and thereafter the residual activity was determined at pH 10.0 and 60°C. Similarly, the temperature stability was determined by incubating the enzyme samples at various temperatures ranging from 40–80°C for different time intervals up to 2(1/2) h.

#### 2.8.2. Effect of Inhibitors and Metal Ions

The effect of various inhibitors namely PMSF, 1,10-phenanthroline, EDTA, N-bromosuccinmide, iodoacetamide, and *β*-mercaptoethanol, DTT (Sigma-Aldrich USA; ICN chemicals, USA) on keratinase activity was carried out by incubating the enzyme with inhibitors at a final concentration of 1 mM at room temperature for 5 min. Similarly, the effect of metal ions was studied by pre-incubating the enzyme with metal ions namely Ba^2+^, Ca^2+^, Cd^2+^, Cr^3+^, Cu^2+^, Hg^2+^, Mg^2+^, Mn^2+^, Ni^2+^and Zn^2+^at a final concentration of 5 mM for 1 h and then determining the residual activity at pH 10.0 and 60°C. The activity was expressed as residual percentage activity against the control without inhibitors which was taken as 100%.

#### 2.8.3. Stability in Presence of Surfactants, Detergents, and Oxidizing Agents

The compatibility of keratinase towards various ionic and nonionic surfactants namely saponin, triton X-100, sodium cholate, SDS, tween-80, and detergents was tested by pre-incubating the enzyme with surfactants/detergents at a final concentration of 1% (w/v) for 1 h at room temperature. Also, its stability towards oxidizing agents like hydrogen peroxide (H_2_O_2_) and sodium cholate (NaHClO_3_) was checked by incubating enzyme with varying concentrations of oxidizing agents for 1 h at room temperature. Similar controls were set up using inactivated enzyme. The keratinase activity was determined at pH 10.0 and 60°C. The activity was expressed as relative activity with respect to control (without any additive) which was taken as 100%.

#### 2.8.4. Substrate Specificity of Keratinase

The substrate specificity of the keratinase was studied by comparing proteolysis of both soluble and insoluble substrates namely azo-casein, BSA, casein, gelatin, haemoglobin, elastin, feather keratin, fibrin, keratin azure, and *α*-keratin (Sigma-Aldrich USA). Twenty mg of each of the substrates was added to 1 mL of appropriately diluted enzyme in glycine-NaOH buffer 100 mM, pH 10.0 and incubated at 60°C for 1 h. The reaction was stopped by adding 4 mL of 5% (w/v) trichloroacetic acid. The contents were centrifuged after 1 h at 7441 g for 10 min. Folin Ciocalteau's reagent (0.5 mL) was added to 1 mL of the supematant, and the optical density of the samples was taken at 660 nm against appropriate substrate and enzyme blanks. One unit of protease was equivalent to the amount of enzyme required to release 1 *μ*g of tyrosine mL^−1^h^−1^under standard assay conditions.

#### 2.8.5. Amidolytic Activity of Keratinase

The purified keratinase was also examined for its ability to hydrolyze synthetic *p*-nitroanilide substrates namely N-Suc-Ala-Ala-Pro-Phe-*p*NA, N-Ala-Ala-Pro-Leu-*p*NA, N-Suc-Ala-Ala-Ala-*p*NA, N-Suc-L-Phe-*p*NA, N-Suc-Gly-Gly-Phe-*p*NA, N-Benzoyl-DL-Arg-*p*NA, and N-Benzoyl-L-tyrosine-*p*NA, N-CBz-L-Phe-*p*NA (Sigma-Aldrich USA; ICN chemicals, USA). Ten mM stock solutions of the peptides were prepared in DMSO. One mM of the substrates was added to appropriately diluted enzyme in glycine-NaOH buffer (100 mM, pH 8) and incubated at 60°C. The reaction mix was incubated at optimum temperature for 10 min. The hydrolyzed product was measured at 405 nm using a UV-Vis spectrophotometer (UV 1700 Shimadzu, Japan). The molar extinction coefficient for *p*NA was taken to be 9900 M^−1^cm^−1^ [10].

#### 2.8.6. Determination of Kinetic Constants

The most effectively hydrolyzed substrates, N-Suc-Ala-Ala-Pro-Phe-*p*NA, N-Ala-Ala-Pro- Leu-*p*NA, and casein were selected for kinetic studies. Varying concentrations of synthetic *p*-nitroanilide substrates namely N-Suc-Ala-Ala-Pro-Phe-*p*NA and N-Suc-Ala-Ala-Pro-Leu-*p*NA (0.1–1.0 mM) and casein (0.5–10 mg/mL) were assayed with 1 mL appropriately diluted enzyme at optimum pH and temperature.

#### 2.8.7. Hydrolysis of Insulin B Chain and Mass Spectrometry

The substrate specificity of keratinase was also studied by hydrolysis of insulin B-chain (Sigma, cysteine residues oxidized). One hundred  *μ*  L of insulin B-chain (1 mg/mL in 10 mM Tris-HCl buffer, pH 9) was mixed with 100 *μ*L of purified enzyme. The mixture was then incubated at 60°C for 16 h. After 16 h of incubation, 40 *μ*L of 0.1% (v/v) TFA was added to the reaction mixture to inactivate the enzyme. Hydrolysis of insulin B chain by enzyme was analyzed by liquid chromatography-electron spray mass spectrometry (LC-ESI/MS, GenPro Biotech, India). Cleavage sites were determined using FindPept software of Expasy.

## 3. Results

### 3.1. Purification of Keratinase

**Figure 1:**
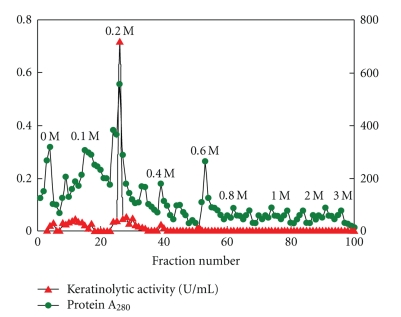
Elution profile of keratinase on DEAE-Sepharose column.

One major keratinase peak was eluted from DEAE-Sepharose column in 0.2 M NaCl fraction (Figure 1). Keratinase was purified with a purification fold of 2.19 and an overall recovery of 92% (Table 1).

### 3.2. SDS-PAGE and HPLC Analysis

**Figure 2:**
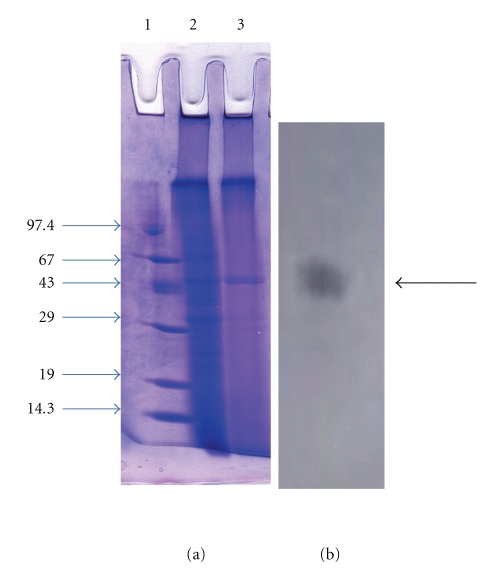
SDS-PAGE and zymogram analysis of purified keratinase (a) SDS PAGE, Lane 1: Protein molecular mass marker, Lane 2: Culture supernatant, Lane 3: Purified fraction eluted from DEAE Sepharose column (b) Zymogram.

**Figure 3:**
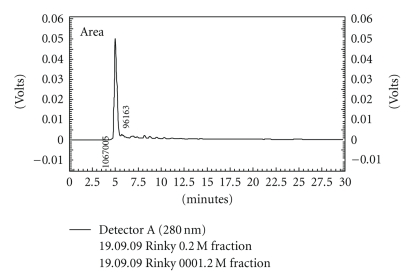
HPLC profile of purified keratinase.

The homogeneity of the purified keratinase was revealed by SDS-PAGE, showing a single band at 45 kDa (Figure 2). Purity was also confirmed by HPLC analysis, where a single major peak having retention time of 4.9 min was obtained on C18 column using UV detector (Figure 3). In zymogram, a clear zone of casein hydrolysis was observed against a white background coinciding with the protein band on the gel (Figure 3).

### 3.3. Effects of pH on the Activity and Stability of Keratinase

**Figure 4:**
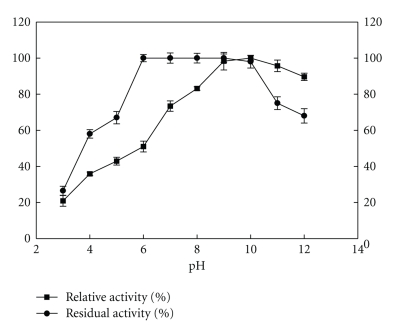
Effect of pH on activity and stability of keratinase.100% activity corresponded to 720 U/mL on feather keratin as substrate.

The enzyme was active in neutral to alkaline pH range from pH 7.0–12.0 with maximum activity corresponding to 720 U/mL at pH 10.0 (Figure 4). It was completely stable in the pH range of pH 6.0–10.0 with no loss in activity and retained up to >50% activity at extreme acidic (pH 4.0-5.0) and alkaline pH (pH 11.0-12.0) after 2 h of incubation at room temperature.

### 3.4. Effect of Temperature on the Activity and Stability of Keratinase

**Figure 5:**
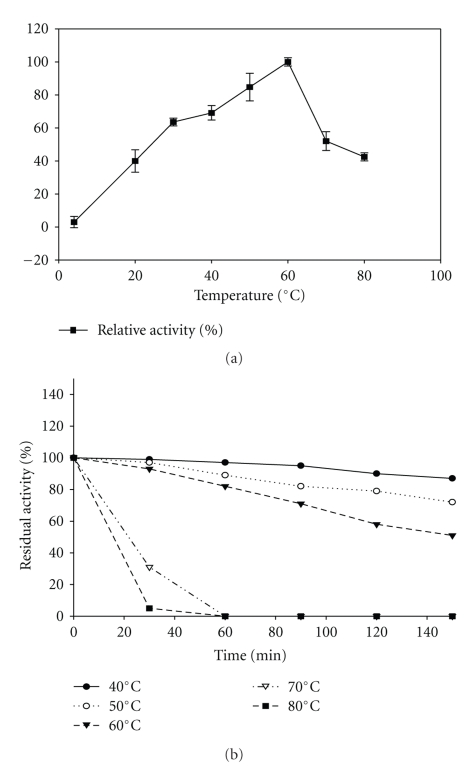
Effect of temperature on the activity of keratinase. 100% activity corresponded to 720 U/ml on feather keratin as substrate.

The enzyme was active over a wide temperature range from 30 to 60°C with maximum activity at 60°C (Figure 5). It was stable over temperatures ranging from 40–60°C with 97% residual activity (699 U/mL) at 40°C followed by 89% activity (642 U/mL) at 50°C while 82% residual activity (591 U/mL) at 60°C after 1 h of incubation at respective temperatures. It exhibited a *t*_1/2_ > 2 h at 60°C, *t*_1/2_ of 30 min at 70°C, and *t*_1/2_ of 20 min at 80°C (Figure 5).

### 3.5. Effects of Inhibitors and Metal-Ions

Among the various inhibitors PMSF completely inhibited keratinase activity followed by 70% residual activity corresponded to 505 U/mL in presence of 10 mM EDTA. It was highly stimulated by the presence of reducing agents like *β*-mercaptoethanol and DTT where twofold activation in presence of 10 mM DTT and eightfold enhancement in 10 mM *β*-mercaptoethanol were observed (Table 2). It retained more than 80% activity in the presence of divalent cations like Ba^2+^, Ca^2+^, Cd^2+^, Cr^2+^, Cu^2+^, Mg^2+^, Mn^2+^, Ni^2+^, and Zn^2+^ after 1 h of incubation at room temperature (data not shown).

### 3.6. Stability in Presence of Surfactants, Detergent and Oxidation

Surfactants like saponin, triton X-100, sodium cholate, SDS, and tween-80 stimulated the activity of keratinase with a maximum activation of twofold in presence of triton X-100 (Table 3). Remarkable was the compatibility of present keratinase towards various commercially available detergents with a stimulation of 1.8-fold (Table 3) and oxidation stability with nearly two-to threefold enhancements in presence of H_2_O_2 _and NaHClO_3_ (Table 4).

### 3.7. Substrate Specificity of Keratinase

**Figure 6:**
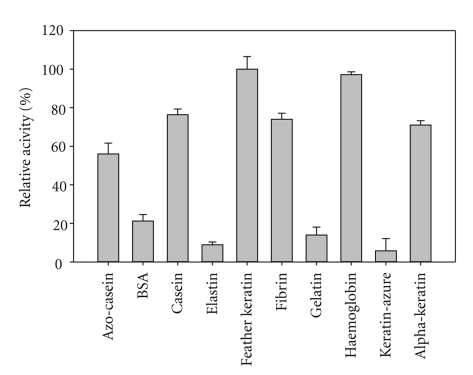
Substrate specificity of keratinase on complex substrates.

**Figure 8:**
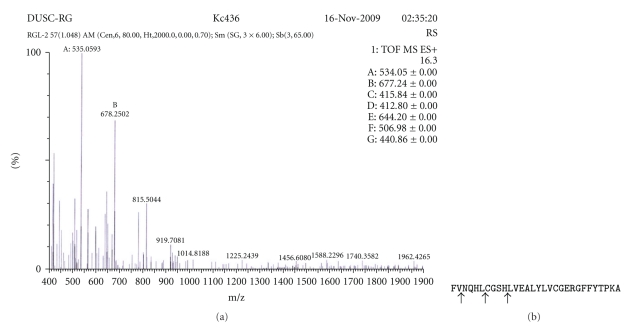
Hydrolysis of insulin B-chains (with oxidized cysteine residues) by purified keratinase. (a) ESI-LC-MS/MS data (b) Sequence of insulin B-chain. Arrows represent cleavage sites.

Keratinase from *B. pumilus* KS12 hydrolyzed most of soluble and insoluble complex substrates with 100% relative activity corresponded to 720 U/mL on feather keratin and 97% activity corresponding to 699 U/mL on haemoglobin followed by >50% relative activity on casein, fibrin, *α*-keratin, and azo-casein while, BSA, gelatin, and elastin were least hydrolyzed with <20% relative activity, respectively (Figure 6). Among synthetic substrates, it hydrolyzed N-Suc-Ala-Ala-Pro-Phe-*p*NA > N-Ala-Ala-Pro-Leu-*p*NA > N-Suc-Ala-Ala-Ala-*p*NA. Kinetic studies revealed a K_m_ of 0.25 mg/mL, 0.1 mM, and 0.25 mM and *V*_max _ of 0.75 *μ*g/mL/min, 2.0 *μ*moles/mL/min, and 3.0 *μ*moles/mL/min on casein, N-Suc-Ala-Ala-Pro-Phe-*p*NA, and N-Ala-Ala-Pro-Leu-*p*NA, respectively (Table 5). LC-ESI/MS analysis of the hydrolyzed insulin B-chain revealed the cleavage sites to be between Val^2^- Asn^3^, Leu^6^-Cys^7^,and His^10^-Leu^11^ (Figure 8).

## 4. Discussion

*Bacillus pumilus* KS12 is a potential feather degrader as it degraded chicken feathers within 24 h at 37°C, 200 rpm. Keratinases from *Bacillus *species and *Streptomyces *species have been extensively studied, however, till date only *Bacillus licheniformis* keratinase has been commercially well established [2]. A 45 kDa monomeric keratinase was purified from the fermentation broth of *Bacillus pumilus* KS12. This is different from the earlier reported keratinases from *Bacillus pumilus *which are of 34 and 65 kDa [11, 12]. 

Keratinase from *Bacillus pumilus* KS12 was an alkaline, serine protease with maximal activity at pH 10. This is in accordance with several earlier reports on microbial keratinases showing pH optima in alkaline range of pH 8 to 11 [2]. However, it was different from the optima of pH 9 and pH8 for keratinase from *Bacillus pumilus* A1 and *Bacillus pumilus* CLRI, respectively [13, 14]. It is assumed that alkaline conditions aid in breaking of disulfide bonds and assists rapid feather degradation. It also had a wide range of pH stability with more than 90% relative activity over a range of pH 6–10.

Keratinases from *Bacillus pumilus* KS12 had temperature optima at 60°C. Most keratinases are thermoactive with temperature optima in the range of 50–60°C with some exceptions such as *Fervidobacterium islandicum* AW-1 keratinase which showed high temperature optima of 90°C and 100°C, respectively [11]. The present keratinase also had high stability more than 80% over a temperature range of 40–60°C with a *t*_1/2_>2 h at 60°C.

It was inhibited by PMSF thus it is a serine protease. This is in confirmation with most of the reported keratinases [2]. Noteworthy was the fact that it was highly thiol activated as its activity was enhanced by two and eight folds in presence of 10 mM DTT and *β*-mercaptoethanol. Thiol activation of keratinases has been reported earlier, and it is a positive attribute for their action on cysteine rich keratin substrates [2]. 

The present enzyme had broad substrate specificity for both soluble and insoluble complex and synthetic substrates. It effectively hydrolyzed most of complex substrates with maximum activity on feather keratin followed by haemoglobin, casein, fibrin, *α*-keratin, azo-casein, and gelatin. Most of the keratinolytic enzymes do have good caseinolytic activity [1]. Hence, K :  C (keratinolytic : caseinolytic) ratio is regarded as a parameter for comparing keratinolytic potential of various proteases, and a protease with ratio of >0.5 is considered as a keratinase [12]. In this perspective, the present enzyme is a potential keratinase with a K : C of 1.5. Besides this, the activity on synthetic substrates and insulin B-chain cleavage also revealed that it preferentially cleaves hydrophobic residues. This again reconfirms its action on insoluble, hydrophobic proteins. By virtue of these properties, it can become a potential catalyst for degradation of insoluble, hard-to-degrade proteins including prion plaques.

Overall, the present keratinase is a thermoactive, alkaline, thiol-activated, oxidation stable enzyme with broad range of substrate specificity and thus can find applications in various industries especially in detergent sector for collar washings to remove scurf and in hospital washings to remove blood stains. 

Further work can be extended towards development of commercially viable enzyme by immobilization on inert insoluble substrates as reported earlier for keratinase from *Bacillus subtilis* on calcium alginate beads [15] and recently on polymer assisted nanoparticles from *Bacillus subtilis* [16] and *Bacillus halodurans* [3].

## Figures and Tables

**Figure 7:**
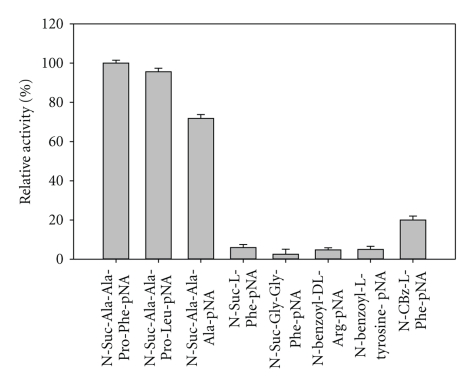
Amidolytic activity keratinase on *p-*nitroanilides.

**Table 1:** Purification scheme of keratinase.

Components	Total enzyme activity (U)	Total protein (mg)	Specific activity (U/mg)	Recovery (%)	Purification fold
Lyophilized Culture broth	2170.00	12.00	180.83	100.00	1.00
DEAE-Sepharose	716.00	1.80	397.77	32.99	2.19

**Table 2:** Effect of inhibitors on activity of keratinase.

S. no.	Concentration (mM)	Residual activity (%)			
		PMSF	EDTA	DTT	*β*-mercaptoethanol
(1)	1	5.00	74.00	120.00	240.00
(2)	5	0.00	70.00	158.00	609.00
(3)	10	0.00	70.00	203.00	876.00

Control without inhibitors was taken as 100% activity corresponded to 720 U/mL on feather keratin as substrate.

**Table 3:** Stability of the keratinase in the presence of various surfactants and detergents.

Surfactants/ Detergents (1% w/v, v/v)	Relative activity (%)
Control*	100.00
Saponin	191.00
Sodium cholate	187.00
SDS	145.00
Triton-X	202.00
Tween-80	140.00

*Detergents*	
Ariel	183.00
Henko	185.00
Fena	180.00
Nirma	183.00
Rin	180.00
Surf	160.00
Tide	179.00
Wheel	183.00

*Control set up with inactivated enzyme was taken as 100% activity corresponded to 720 U/mL on feather keratin as substrate.

**Table 4:** Effect of oxidizing agents on activity of keratinase.

S. no.	Concentration	Relative activity (%)	
	(%)	H_2_O_2_	NaHClO_3_
(1)	1	328.08	257.0
(2)	2	350.0	226.0
(3)	3	363.0	226.0

Control set up with inactivated enzyme was taken as 100%.

**Table 5:** Kinetic parameters of purified keratinase on casein and *p*NA.

Substrates	K_m_	*V*_max _	K_cat_ (min^−1^)
Casein	0.25 mg/mL	0.75 *μ*g/mL/min	3.0
N-Suc-Ala-Ala-Pro-Phe-pNA	0.1 mM	2.0 *μ*moles/mL/min	20.0
N-Ala-Ala-Pro-Leu-pNA	0.25 mM	3.0 *μ*moles/mL/min	12.0
